# Supplementary figures and images for: Claw shape variation in oribatid mites of the genera Carabodes and Caleremaeus: exploring the interplay of habitat, ecology and phylogenetics
Source: PeerJ. 2023 Sep 25;11:e16021. doi: 10.7717/peerj.16021 (PMC10538281; doi:10.7717/peerj.16021)

A)

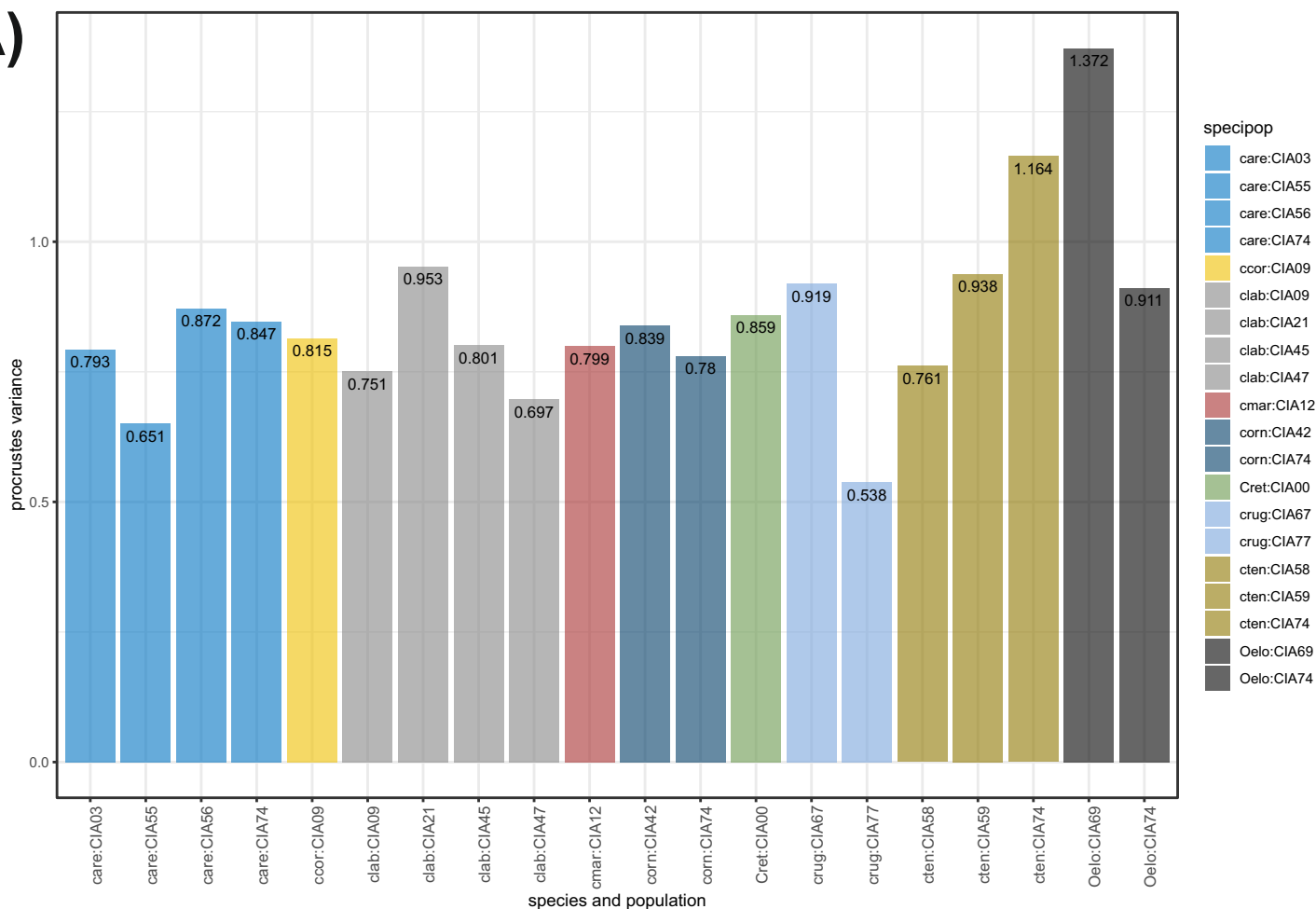

B)

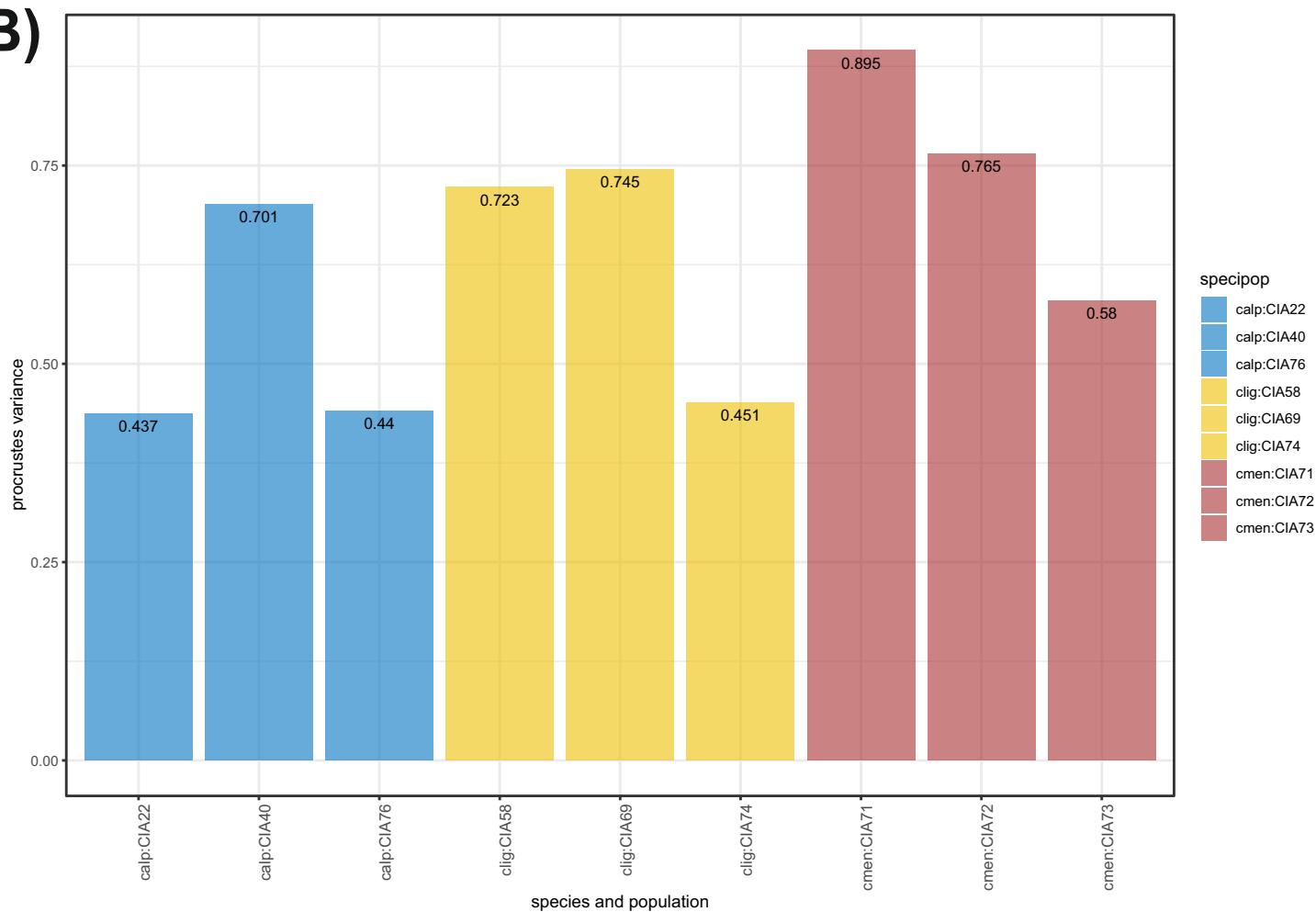

Supplement: Figure S1 — Claw disparity (procrustes variance) in Carabodes and Caleremaeus species [file peerj-11-16021-s001.pdf]

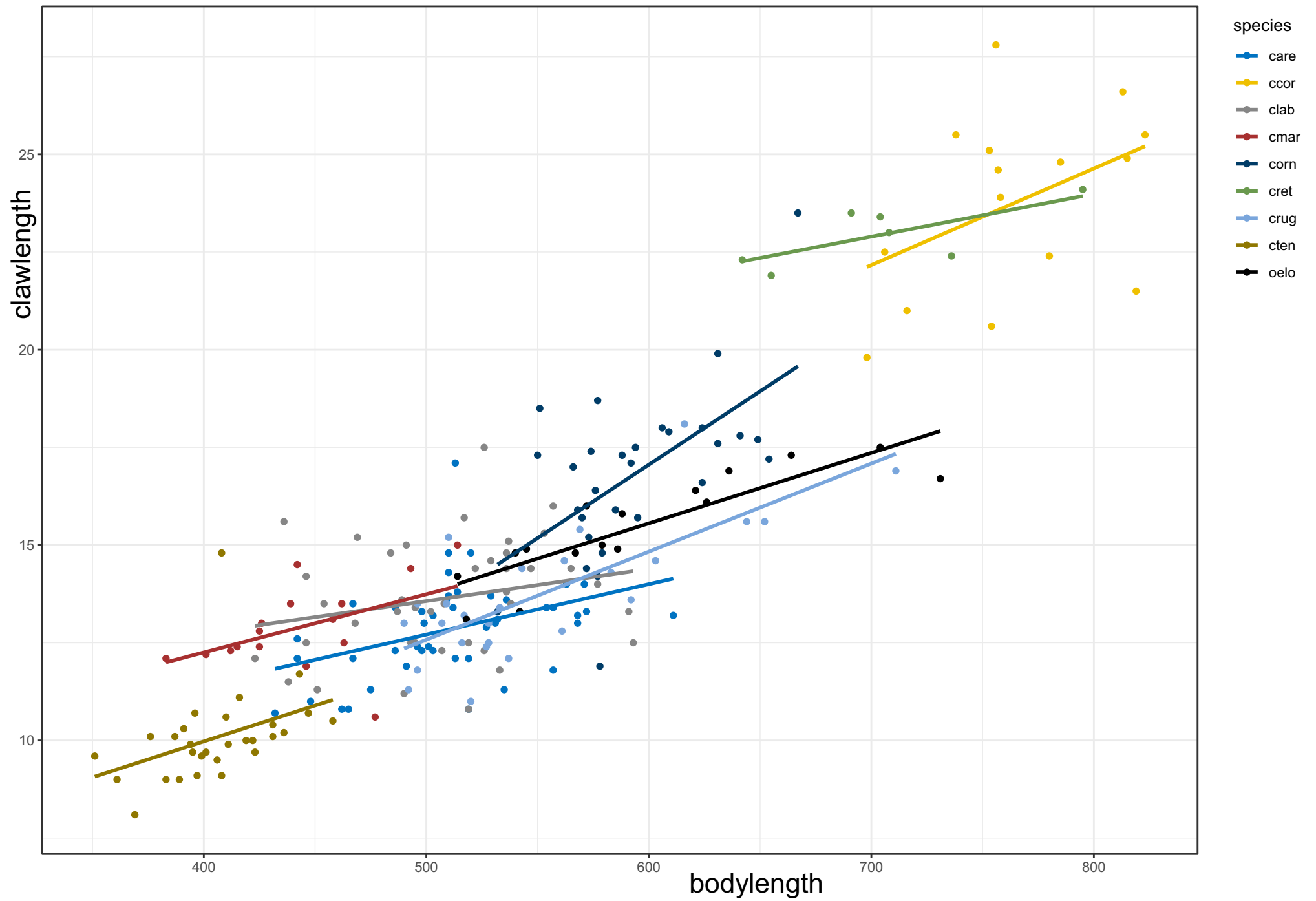

Supplement: Figure S2 — Clawsize in relation to bodylength in different Carabodes species. [file peerj-11-16021-s002.pdf]

gamma

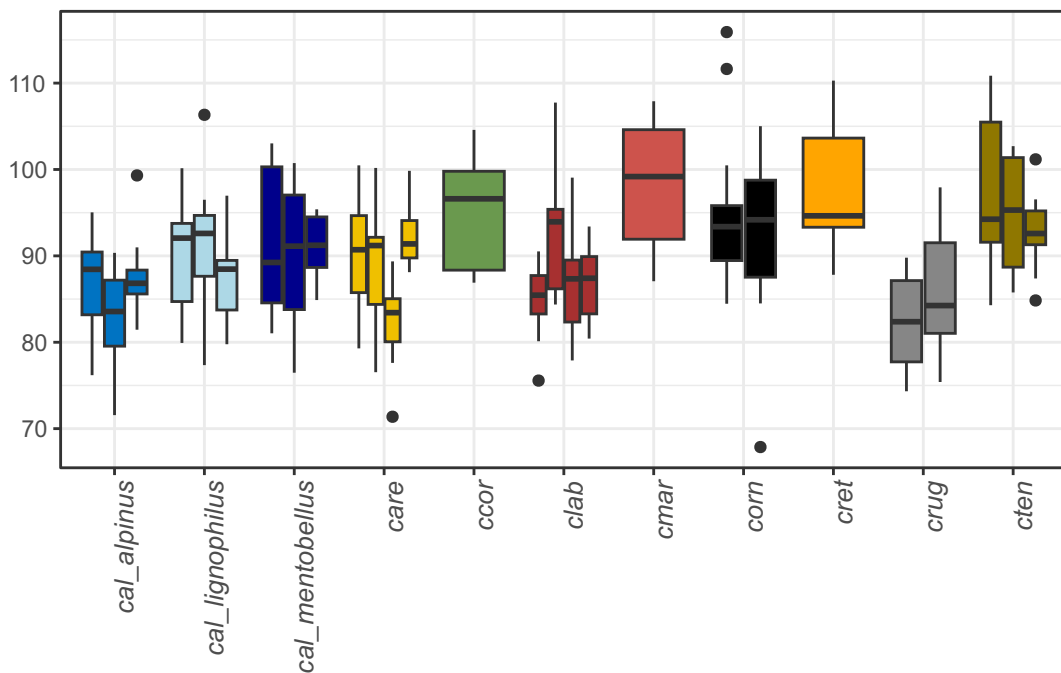

clawlength

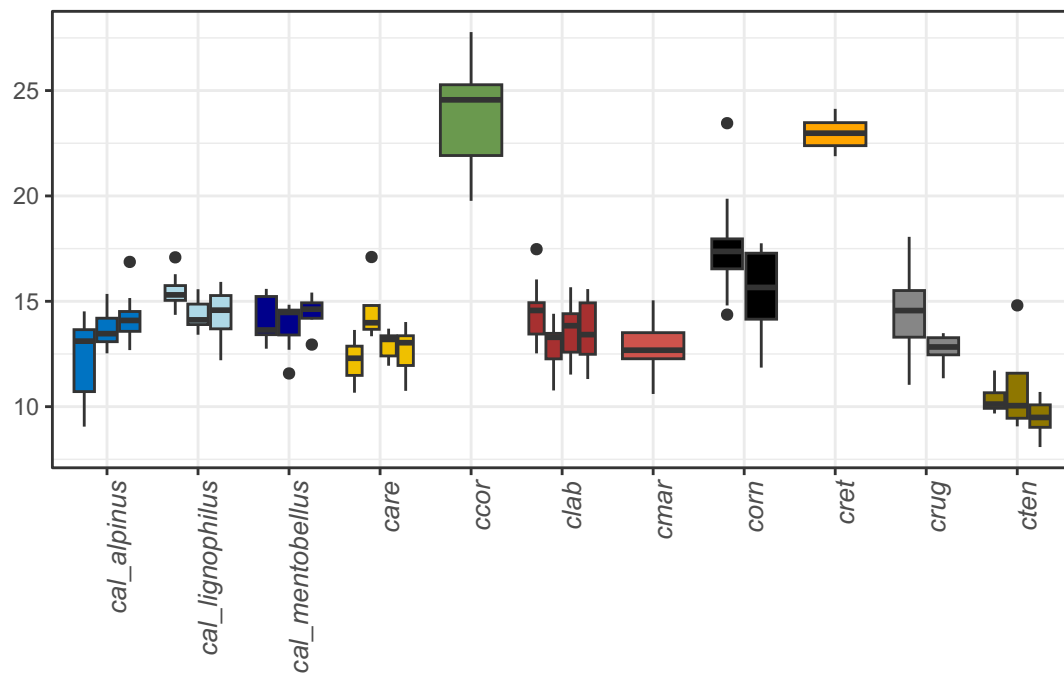

cl/bL

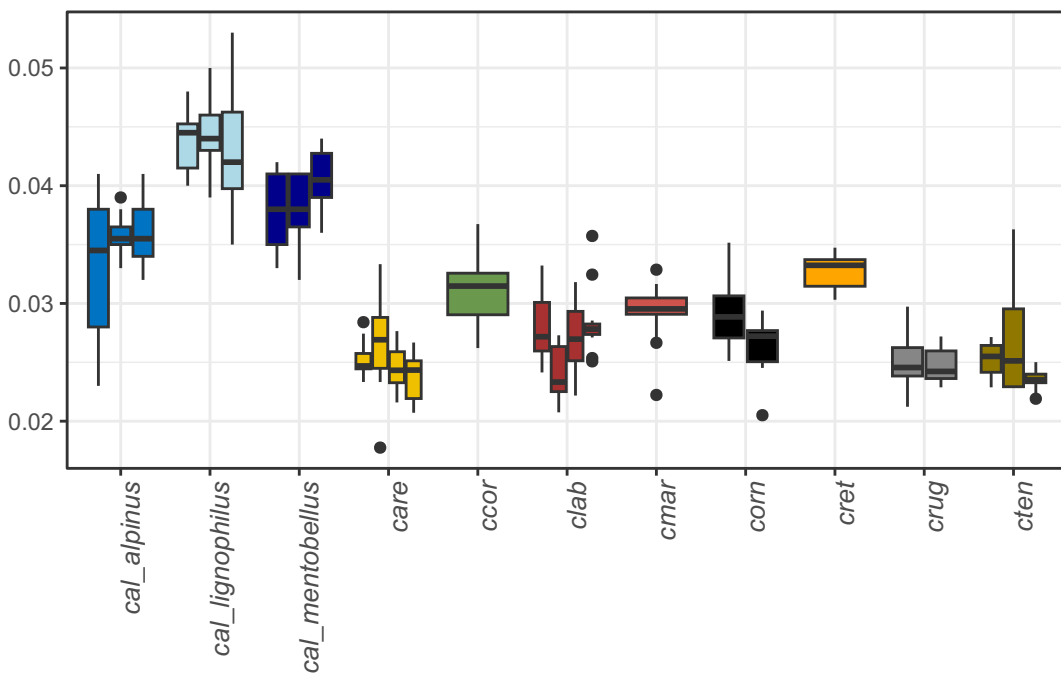

bodylength

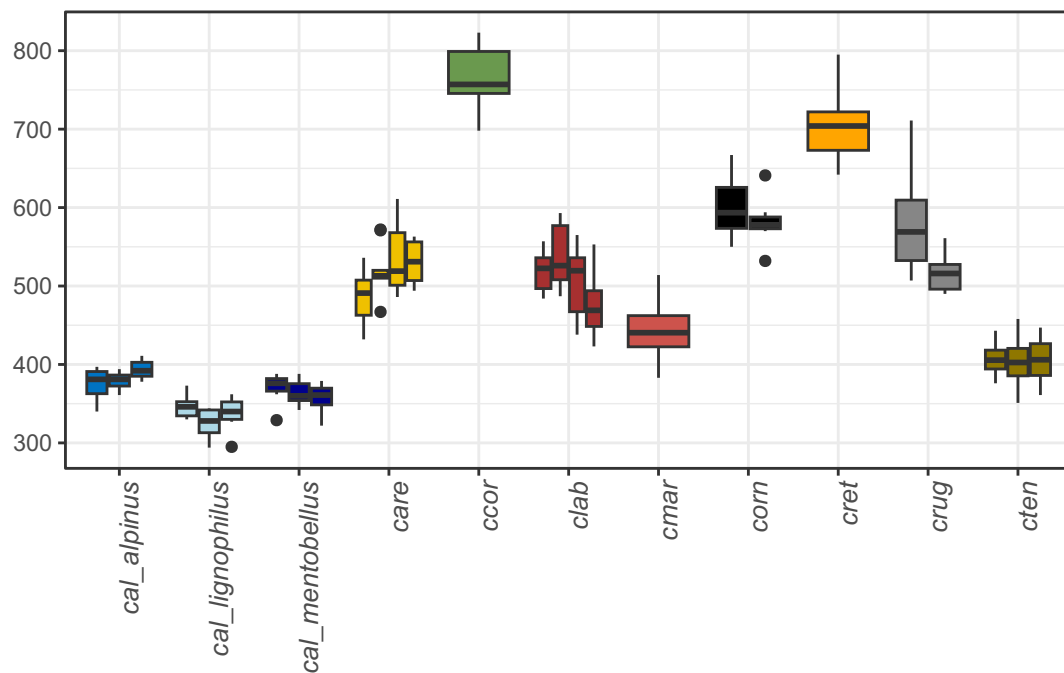

Supplement: Figure S3 — Measurements in populations and species of both genera. [file peerj-11-16021-s003.pdf]

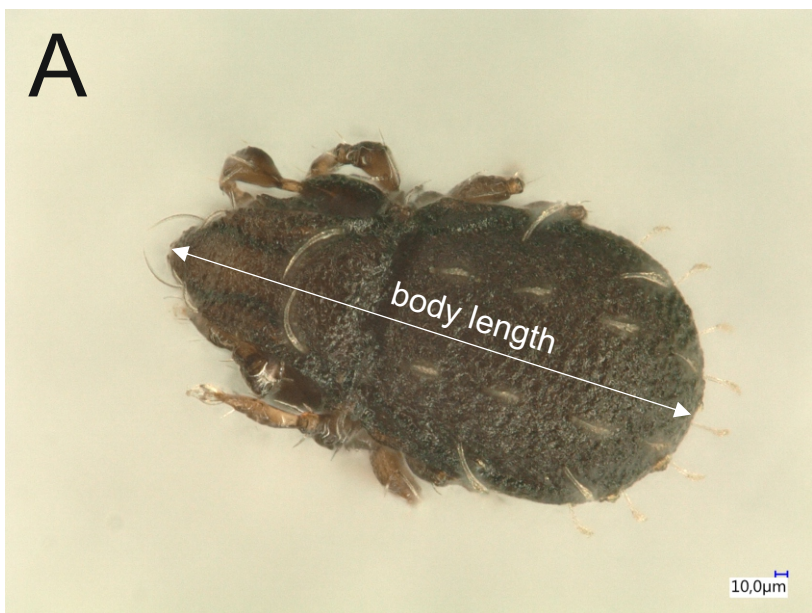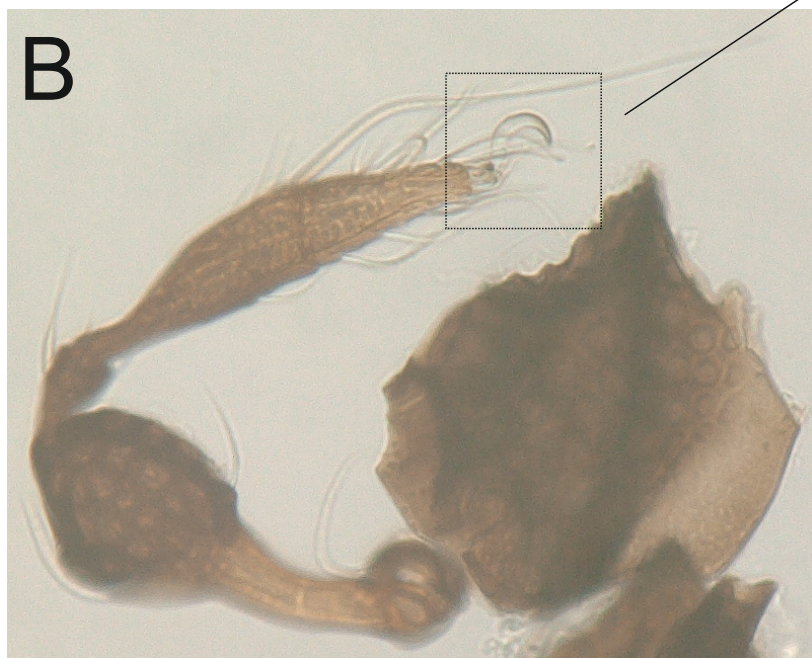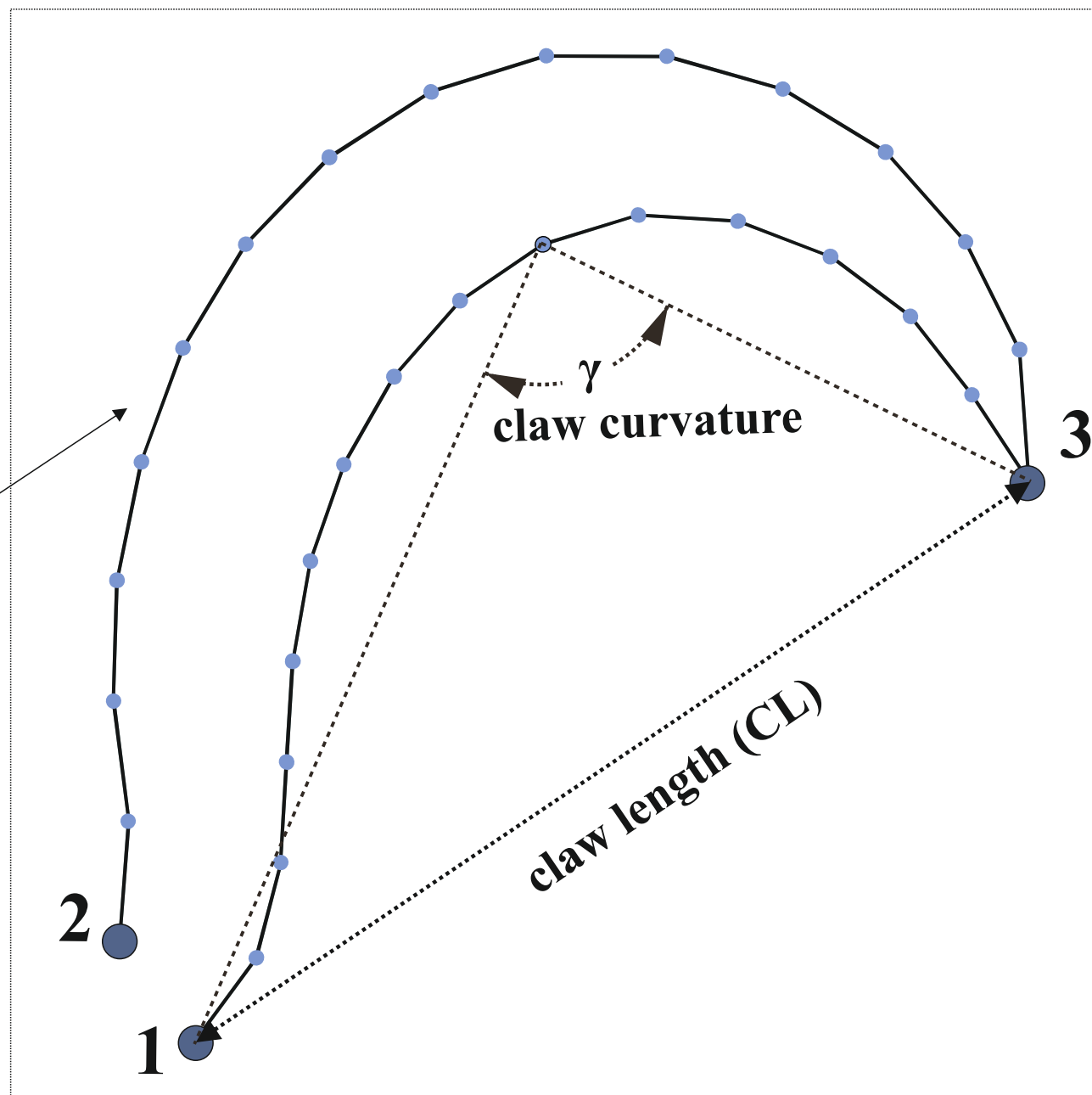

Supplement: Figure S4 — (A) Dorsal view of Carabodes areolatus with body length measurement and (B) photograph of the first leg and schematic drawing of the claw with landmarks, semilandmarks and measurements. [file peerj-11-16021-s004.pdf]
